# Age at mating and male quality influence female patterns of reproductive investment and survival

**DOI:** 10.1002/ece3.5137

**Published:** 2019-04-08

**Authors:** Kerianne M. Wilson, Sean E. Walker

**Affiliations:** ^1^ Department of Ecology and Evolutionary Biology University of California Irvine California; ^2^ Department of Biological Sciences California State University Fullerton California

**Keywords:** aging, house crickets, life history, mate quality, sexual conflict

## Abstract

The trade‐off between the allocation of resources toward somatic maintenance or reproduction is one of the fundamentals of life history theory and predicts that females invest in offspring at the expense of their longevity or vice versa. Mate quality may also affect life history trade‐offs through mechanisms of sexual conflict; however, few studies have examined the interaction between mate quality and age at first mating in reproductive decisions. Using house crickets (*Acheta domesticus*), this study examines how survival and reproductive trade‐offs change based on females’ age at first reproduction and exposure to males of varying size. Females were exposed to either a large (presumably high‐quality) or small male at an early (young), middle (intermediate), or advanced (old) age, and longevity and reproductive investment were subsequently tracked. Females mated at a young age had the largest number of eggs but the shortest total lifespans while females mated at older ages produced fewer eggs but had longer total lifespans. The trade‐off between age at first mating and eggs laid appears to be mediated through higher egg‐laying rates and shorter postmating lifespans in females mated later in life. Exposure to small males resulted in shorter lifespans and higher egg‐laying rates for all females indicating that male manipulation of females, presumably through spermatophore contents, varies with male size in this species. Together, these data strongly support a trade‐off between age at first reproduction and lifespan and support the role of sexual conflict in shaping patterns of reproduction.

## 1 INTRODUCTION

Individuals have limited resources to allocate toward either reproduction or somatic maintenance, which results in a trade‐off between survival and reproduction (Kirkwood & Rose, 1991). For example, individuals may invest in reproduction early in life at the cost of longevity, or individuals may wait to reproduce, leaving less time and potentially resources for reproduction but living longer. Thus, life history theory suggests that females that begin reproducing early in life have a fitness advantage over those that postpone reproduction (Roff, 2002; Stearns, 1989,1992). Yet, females—even in short‐lived species—vary in their timing of reproduction. While environmental circumstances, such as poor nutritional conditions, may arise in which it is advantageous for females to postpone reproduction (Bertness, 1981; Moehrlin & Juliano, 1998; Wessels, Kristal, Netter, Hatle, & Hahn, 2011), other factors, such as the availability of preferred or high‐quality mates, may also play a role in a female's decision to postpone mating. Given the trade‐off between reproduction and longevity, it is unclear whether postponing reproduction in hopes of locating a better mate is an advantageous strategy since the fitness benefits from mating with a high‐quality male may or may not outweigh the fitness costs associated with initiating offspring production later in life. The extent to which male characteristics and the timing of female reproduction may interact to impact investment patterns and ultimately, fitness, have not been thoroughly explored.

There is ample evidence that females can adjust reproductive investment patterns in response to male quality (Burley, 1988; Gowaty, 2008; Kindsvater, Rosenthal, & Alonzo, 2013; Sheldon, 2000; Ting, Judge, & Gwynne, 2017). However, it remains unclear how female age influences these decisions. Since females that postpone reproduction (older females) have fewer reproductive opportunities and are more constrained than those that begin producing offspring earlier (young females), reproductive allocation patterns are expected to vary across an individual's lifetime (Cotter, Ward, & Kilner, 2010; Harris & Uller, 2009; McNamara & Houston, 1996; McNamara, Houston, Barta, Scheuerlein, & Fromhage, 2009; Roff & Gelinas, 2003). Young females have more opportunities to find mates and are more selective of mates (Gray, 1999; Mautz & Sakaluk, 2008) suggesting, as predicted by life history theory, that young females possess higher reproductive value than older females. It is expected that young females will also be more selective about the allocation of their reproductive resources (Gowaty & Hubbell, 2009). In contrast, older females should be less selective and invest at a greater rate and potentially more, both in terms of offspring size and number, as they age if their condition permits (Kindsvater, Alonzo, Mangel, & Bonsall, 2010). However, larger investments over shorter time periods are costly. This strategy of quickly investing significant resources toward reproduction is expected to reduce somatic allocation, which may impact postmating female survival resulting in less time to invest in reproduction. Thus, it is unclear whether females can compensate for postponed investment in fitness or whether there may be other consequences such as a trade‐off between fecundity and fertility. These factors are likely to inform age‐specific trade‐off patterns observed for house crickets (*Acheta domesticus*).

Despite an expected decrease in offspring quantity, postponing reproduction in order to find a higher‐quality mate may confer benefits via offspring quality. For example, high‐quality male house crickets can confer heritable benefits to offspring (Ryder & Siva‐Jothy, 2001), thus, a female may choose to wait to reproduce and attempt to find a better mate. This may be especially true in species with lek‐style mate systems like crickets since variation in female fitness across generations will be largely dependent on the success of her sons. Thus, mate choice decisions should be associated with indirect benefits (Endler & Basolo, 1998; Hunt & Sakaluk, 2014; Prokop, Michalczyk, Drobniak, Herdegen, & Radwan, 2012).

Previous studies have established that female house crickets prefer larger males (Gray, 1997; Ryder & Siva‐Jothy, 2001; Stoffer & Walker, 2012). For this species, it is known that immune response and body size are heritable (Head, Hunt, & Brooks, 2006; Ryder & Siva‐Jothy, 2001) and positively correlated (Ryder & Siva‐Jothy, 2001), suggesting that large size is an indicator of male quality. The benefit of a better immune response is clear; however, this may not be the only potential benefit associated with mating with a larger male.

In addition to indirect benefits, mate quality may also be linked to direct benefits that influence reproductive allocation patterns. Since male house crickets to do not provide parental care or nuptial gifts, direct benefits would be conferred through spermatophore contents. Previous studies in crickets have shown that spermatophore contents correlate with male phenotype. For example, in the variable field cricket (*Gryllus lineaticeps*), males with higher chirp rates transfer more sperm to females (Wagner & Harper, 2003). Seminal fluids may also provide benefits or potentially costs to females through compounds such as sex peptides or prostaglandins, which may play a role in sexual conflict. Prostaglandins transferred in house cricket spermatophores induce oviposition (Larson, Andrés, & Harrison, 2012; Loher & Edson, 1973; Murtaugh & Delinger, 1985) and are associated with increased fecundity (Tanaka, 2014; Worthington, Jurenka, & Kelly, 2015) but are also associated with a reduction in female lifespan (Green & Tregenza, 2009; Tanaka, 2014). While a relationship between male attractiveness and amount of prostaglandin transferred during mating has not, to the author's knowledge, been established in house crickets, this relationship has been demonstrated in insects (Pitnick & García‐González, 2002), suggesting prostaglandin levels may vary with other male traits in house crickets as well.

Male characteristics can clearly impact reproductive allocation patterns (Bretman, Rodríguez‐Muñoz, & Tregenza, 2006; Harris & Uller, 2009; Head et al., 2006; Iglesias‐Carrasco, Jennions, Zajitschek, & Head, 2018; Kindsvater & Alonzo, 2014; Ting et al., 2017). Changes in allocation patterns as a function of mate quality have been discussed as differential allocation when females invest more when mated with high‐quality males or reproductive compensation when females invest more when mated with low‐quality males. Although, more recent work has considered them as ends of a continuum (Haaland, Wright, Kuijper, & Ratikainen, 2017; Harris & Uller, 2009; Kindsvater & Alonzo, 2014; Ratikainen & Kokko, 2010). These effects can manifest as changes in fecundity, offspring characteristics, or both. For example, female field crickets (*Gryllus pennsylvanicus*) exposed to songs from high‐quality males and mated to randomly chosen male laid more and larger embryos compared to females exposed to songs from low‐quality males and then, mated with a randomly chosen mate (Ting et al., 2017).

We sought to examine the interaction between age‐specific changes in allocation patterns and mate quality in *A. domesticus* by manipulating age at first reproduction and the size of the male a female was exposed to (and mated with) for 24 hr. We hypothesized that female crickets would adjust reproductive investment based on when they began reproduction and that their exposure to a large or small male was likely to impact investment patterns. We predicted that females mated at younger ages would produce more offspring and have shorter lifespans than those that mated at more advanced ages and that, consistent with differential allocation, females mated with large males would produce more offspring and also have longer lifespans than females mated with small males. Lastly, we expected females mated at more advanced age to have higher rates of reproductive investment and shorter postmating lifespans.

## 2 METHODS

This study was conducted in accordance with the Institutional Animal Care and Use Policies of California State University Fullerton. Juvenile house crickets were obtained from Fluker Farms (Port Allen, LA, U.S.A.) and housed communally in 84 L bins until they reached their adult stage. Bins were checked daily and all adult crickets were removed and housed individually in clear plastic containers (13 × 13 × 4 cm). Crickets were kept on a reverse 12:12 hr light–dark cycle at 25°C. They were maintained on ad libitum cat food and water throughout the study. Since crickets are nocturnal, all subsequent work was conducted during the dark portion of the light–dark cycle under red lighting. Males and females were not acoustically isolated because female house cricket mate decisions are not affected by population density or age (Tinghitella, 2014).

Female age at first mating was selected based on the range of house cricket lifespans for a laboratory setting (Murtaugh & Delinger, 1985; Tinghitella, 2014) and the authors’ past observations of young female mating behaviors. At the time of their male exposure trial, females mated at young (*N* = 59), intermediate (*N* = 25), and old (*N* = 41) ages were 14–16, 24–26, and 34–36 days past adult eclosion, respectively. We henceforth refer to these groups as young, intermediate, or old. Young, intermediate, or old virgin female house crickets were mated with virgin males that were categorized as either large (*N* = 64) or small (*N* = 61) for a total of six treatment groups. Large males weighed more than 0.34 g ($\bar{x}$ ± *SD*=0.39 + 0.03) and small males weighed less than 0.30 g ($\bar{x}$ ± *SD*=0.26 + 0.04) based on previously established female preferences (Stoffer & Walker, 2012). Males selected for the study were all 14–16 days past adult eclosion at the time of mating (males will court females and transfer spermatophores upon reaching their adult molt [e.g., Fleischman & Sakaluk, 2004; Stoffer & Walker, 2012]). Females were randomly assigned to treatment groups, and all individuals were used only once.

At the beginning of each exposure, one male and female were placed in a clean clear enclosed plastic arena (36 × 23 × 17 cm). Each cricket was initially held in a separate smaller container (11 cm diameter, 20 cm height) within the arena for two minutes before being released. Each pair was observed for an hour to ensure normal male courtship behaviors followed by a 23‐hr unobserved exposure period in order to standardize the amount of time each female was exposed to a male. During this time, all pairs had access to food and water. After each exposure, the female was placed back in her original container and the male was euthanized. Females were given a Petri dish containing moist sand and vermiculite in which to lay eggs, which was replaced every two days. Half of the contents of each collected Petri dish were immediately removed and the eggs were counted to ensure oviposition. Since females readily lay eggs after the successful transfer of a spermatophore (Murtaugh & Delinger, 1985; Engelmann, 2015) and unmated females lay few to no eggs (Clifford & Woodring, 1986; Murtaugh & Delinger, 1985), females who did not lay eggs in the first 2 days post male exposure and those that did not produce fertile eggs were assumed to have not successfully mated and were not used in the study. The Petri dishes with the remaining eggs, sand, and vermiculite were placed into an incubator for 14 days at 28°C allowing the eggs to develop and hatch. The number of hatchlings, shells, and unhatched eggs were counted. The developmental status (whether or not an egg showed development) was also noted for each egg in order to track fertility. Measurements continued for the lifespan of each female. Since variation in reproductive investment may be observed as differences in primary reproductive allocation (fecundity) and/or fertility, each of these measures of reproductive success was analyzed in order to understand evolutionary consequences of postponed female reproduction and male size. In addition to reproductive measures, total adult lifespan and postmating lifespan were also recorded. Since seminal fluids can influence egg production for up to a week postmating (Worthington et al., 2015), egg‐laying rate was calculated as the number of eggs laid in the first six days after male exposure. Fertilization rate was measured as the proportion of eggs incubated that hatched or showed development. Lifetime egg production and egg‐laying rate were based on total counts of eggs from both incubated eggs and those counted immediately upon collection. Fertilization rates were based on counts from only eggs that were incubated. A combined total of 125 exposure trials are included in the following analyses (young female/small male = 30; young female/large male = 29; intermediate female/small male = 14; intermediate female/large male = 11; old female/small male = 21; old female/large male = 20).

Generalized linear models (ANOVA or logistic regression) were used to analyze the effects of female age at mating and male size on the following response variables: lifespan, postmating lifespan, lifetime egg production, egg‐laying rate (proportion of eggs laid during the first six days) (logistic regression), and fertilization rate (logistic regression). We started with models including all main effects and interactions and simplified the models by removing nonsignificant interactions until only main effects remained. *α* < 0.05 was considered significant for all analyses. Deletion tests for interactions are presented. We evaluated assumptions using diagnostic plots and analyses were carried out using R v 3.5.1. Diagnostic plots for logistic regression (generalized linear models) were examined using the DHARMa package which was also used to test for overdispersion (Hartig, 2019). During examination of diagnostic plots, we checked for outliers as well as other assumptions (e.g., normality for ANOVA). Logistic regression models were fit using glm in R. In both cases, the data were overdispersed and to account for this we used the quasibinomial error distribution instead of the binomial error distribution (Crawley, 2013). We controlled for family size error rate when doing multiple comparisons using the Holm procedure (Holm, 1979). The following variables were transformed in order to meet the assumptions of ANOVA: lifetime egg production (square root) and total and postmating life span (log). Since lifetime egg production is correlated with female mass, we performed analyses of this response variable using female mass as a covariate. Although female mass was correlated with the response variable, it did not change our results so these analyses are not presented.

## 3 RESULTS

**Figure 1:**
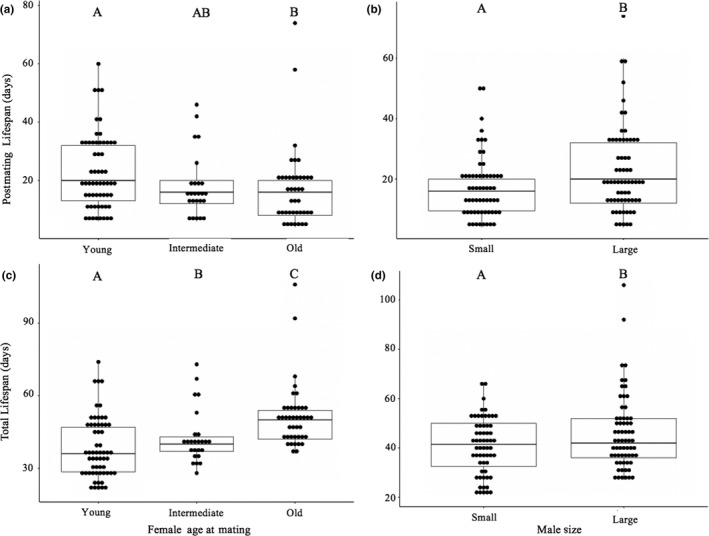
Number of days females lived postadult eclosion and postmating. Postmating and adult lifespan significantly differed based on female age at mating (a and c) and mate size (b and d). Letters indicate significant differences among treatments as determined by the Holm procedure. Results correspond to Table 1. The box represents the 25th, 50th (median), and 75th quartile. The whiskers are 1.5 × interquartile range (IRQ). Whiskers show the maximum of the data if it is <1.5 × IQR

Female age at first mating and male size influenced female postmating lifespan and total female lifespan (Table 1). Total female lifespan differed significantly among females mated at different ages with young females having the shortest total lifespans (*p* ≤ 0.039) and old females having the longest (*p* ≤ 0.018) (Figure 1a,c). Old females had shorter postmating lifespans than young females (*p* = 0.021). Females exposed to large males had longer postmating and total lifespans than females exposed to small males (Figure 1b,d).

**Table 1:** Female lifespan

Variable	Degrees of freedom	Sum of squares	Mean square of the error	*F*‐value	*p*‐Value
Postmating lifespan	5, 124			2.83	0.019
Female age at mating	2	2.92	1.46	4.12	0.019
Male size	1	2.08	2.08	5.89	0.017
Residuals	121	42.78	0.35		
Total lifespan	5, 124			7.00	<0.0001
Female age at mating	2	2.44	1.22	17.15	<0.0001
Male size	1	0.39	0.39	5.50	0.021
Residuals	121	8.60	0.07		

Note

Female age at mating (young, intermediate, old) and male exposure (small, large) impacted female postmating lifespan and total lifespan (two‐way ANOVA). The first row for each characteristic is the test for the full model (main effects + interactions). The interactions between female age at mating and male exposure was not significant (postmating lifespan: *F*_2, 119_ = 0.111, *p* = 0.895; total lifespan: *F*_2, 119_ = 0.309, *p* = 0.735).

**Figure 2:**
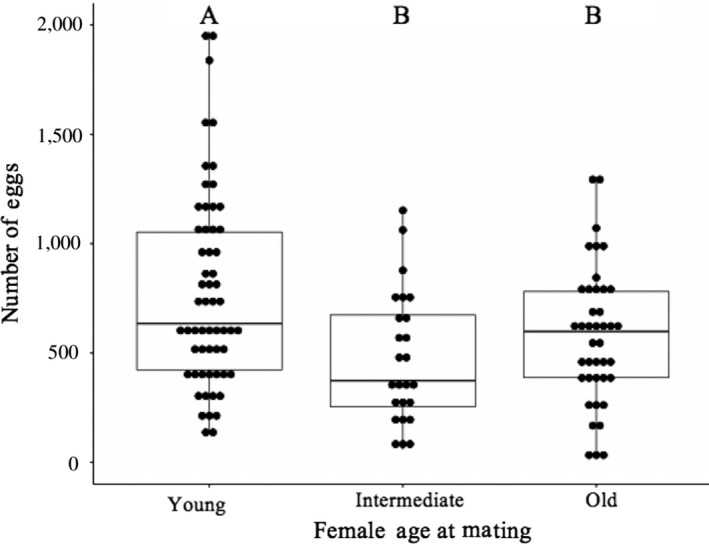
Lifetime production of eggs. The number of eggs laid in a female's lifetime differed significantly based on female age at mating. Letters indicate significant differences among treatments as determined by the Holm procedure. Results correspond to Table 2. The box represents the 25th, 50th (median), and 75th quartile. The whiskers are 1.5 × interquartile range (IRQ). Whiskers show the maximum of the data if it is <1.5 × IQR

**Figure 3:**
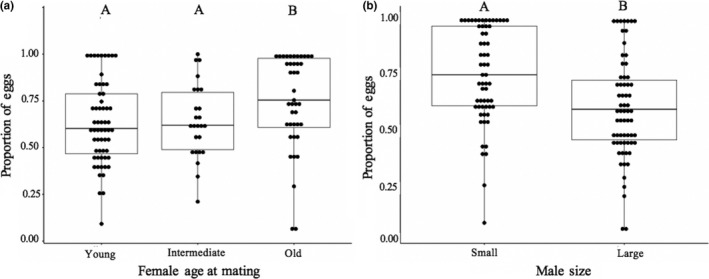
Proportion of the total eggs laid in the first 6 days following male exposure (egg‐laying rate). Egg‐laying rate differed significantly based on female age at mating (a) and mate size (b). Letters indicate significant differences among treatments as determined by the Holm procedure. Results correspond to Table 3. The box represents the 25th, 50th (median), and 75th quartile. The whiskers are 1.5 × interquartile range (IRQ). Whiskers show the maximum of the data if it is <1.5 × IQR

**Figure 4:**
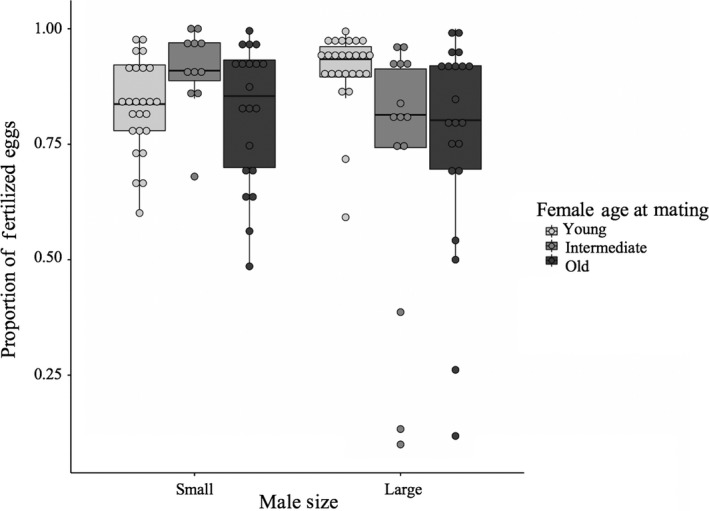
Proportion of total eggs laid that developed including both hatched and unhatched offspring. Fertilization rate tended to differ based on female age at mating and the interaction between female age at mating and male size. Results correspond to Table 3. The box represents the 25th, 50th (median), and 75th quartile. The whiskers are 1.5 × interquartile range (IRQ). Whiskers show the maximum of the data if it is <1.5 × IQR

Female age at first mating influenced the total number of eggs laid and egg‐laying rates (Tables 2 and 3). Young females produced the most eggs (*p* ≤ 0.032) (Figure 2). Male size did not significantly impact the number of eggs produced by females ($\bar{x}$ ± *SD*: small = 539.42 ± 352.77; large = 707.26 ± 429.61; Table 2). Old females had the highest egg‐laying rates (*p* ≤ 0.05) (Figure 3a), and females mated to large males had lower egg‐laying rates than females mated to small males (Figure 3b). There was no significant effect of female age at first mating, male size, or their interaction when examining fertilization rate (which included eggs that hatched and those that showed development; Table 3, Figure 4).

**Table 2:** Lifetime egg production

Variable	Degrees of freedom	Sum of squares	Mean square of the error	*F*‐value	*p*‐Value
Total egg production	5, 124			3.70	0.004
Female age at mating	2	777.65	388.82	6.81	0.002
Male size	1	145.68	145.68	2.55	0.113
Residuals	121	6,910.55	57.11		

Note

Female age at mating (young, intermediate, old) impacted the total number of eggs laid produced in a female's lifetime. The first row is the test for the full model (main effects + interactions). The interaction between female age at mating and male size was not significant *F*_2, 119_ = 1.14, *p* = 0.3241.

**Table 3:** Egg‐laying rate and fertilization rate

Variable	Degrees of freedom	Deviance	Residual degrees of freedom	Residual deviance	*F*‐value	*p*‐Value
Egg‐laying rate	5, 119				5.31	0.0002
Female age and mating	2	509.76	120	3,083.24	10.94	<0.0001
Male size	1	209.64	119	2,873.61	9.00	0.003
Null			122	3,593.01		
Fertilization rate	5, 111				2.54	0.033
Female age and mating	2	30.85	114	674.56	2.90	0.059
Male size	1	4.46	113	670.10	0.84	0.362
Female age and mating × male size	2	31.83	111	638.28	2.99	0.054
Null			114	425.09		

Note

Egg‐laying rate was measured as the number of eggs laid during the 6 days after male exposure and fertilization rate was measured as the proportion of developed eggs relative to total eggs laid. These data were modeled with generalized linear models using a quasibinomial error structure and a logit link. The first row for each characteristic is the test for the full model (main effects + interactions). Female age at mating (young, intermediate, old) and male exposure (small, large) impacted egg‐laying rate. The two‐way interaction between female age at mating and male size was not significant (*F*_2, 117_ = 0.44, *p* = 0.65). Female age at mating (young, intermediate, old) and the interaction between female age at mating and male exposure (small, large) tended to impact the fertilization rate of female eggs.

## 4 DISCUSSION

Patterns of reproductive and somatic investment among females mated at different ages support the trade‐off between survival and reproduction predicted by life history theory (Kirkwood & Rose, 1991; Roff, 2002; Stearns, 1989,1992) in which females that invest more in reproduction have shorter lifespans and females that invest less in reproduction live longer. Females mated at a young age laid more eggs at a lower rate and had shorter lifespans while the opposite pattern was observed for females mated after 25 days (intermediate and old females), which is consistent with classic life history trade‐offs (Roff, 2002; Stearns, 1992). These patterns additionally indicate that postponing reproduction impacts female fitness, which is consistent with studies of senescence demonstrating that reproductive investment declines with age (Flatt & Partridge, 2018; Kirkwood & Rose, 1991).

House crickets clearly exhibit age‐specific plasticity in reproduction. Young female house crickets produced the most eggs while deferred reproduction to an old age was associated with longer total life span and fewer eggs. Postmating lifespans were longer for females mated at young ages than for females mated at older ages and this is inversely correlated with egg‐laying rate since older females laid eggs more quickly than younger females. Taken together, it is clear that shorter total lifespans, but longer postmating lifespans allow for a greater investment in reproduction, while longer total lifespans, but shorter postmating lifespans allow for a more rapid investment in reproduction. Similar patterns have been observed in a number of insects (Evenden, Lopez, & Keddei, 2006; Kaitala, 1991; Lyn, Aksenov, LeBlanc, & Rollo, 2012; Miyatake, 1997; Rogers & Marti, 1996; Travers, Garcia‐Gonzalez, & Simmons, 2015; Wagner & Harper, 2003, but see: Maklakov, Kremer, & Arnqvist, 2007) which suggests a strong adaptive value for maintaining age‐specific plasticity in resource allocation.

Oogenesis of unmated female house crickets continues at least through 18 days postadult eclosion at an increasingly slower rate with age (Clifford & Woodring, 1986). Thus, older females should have more stored eggs in which to oviposit once mated. Why this trend was not observed for intermediate females is unclear and suggests that age‐specific egg storage cannot fully explain how female reproductive investment varies with age. Old females should benefit from rapid egg production even at the cost of longevity given (a) that it is costly to store eggs over a long period both in terms of lost subsequent egg production and reduced gut size (Clifford & Woodring, 1986) and (b) that they are less likely to obtain additional matings compared to younger females. A more complete mechanistic explanation requires further study since very few studies—proximate or ultimate—have considered the effect of postponed mating on life history trade‐offs and even fewer have considered the full range of female age. This area of study will surely provide exciting insights and help underscore the physiological trade‐offs driving age‐specific variations in reproductive strategy described here.

Interestingly, the number of eggs produced by females mated at intermediate and old ages did not differ significantly, but old females laid eggs at a higher rate. This suggests that postponing reproduction past a certain point may result in reduced fitness likely due to lost opportunities for oogenesis and oviposition since old females were unable to fully compensate for fecundity losses by increasing their rate of egg laying, which likely contributed to their reduced postmating lifespan. The difference in reproductive investment patterns between intermediate and old females supports the profitability of assessing female age at more than two levels and highlights nuanced age‐specific trade‐offs that have not been widely considered.

The mechanisms mediating female age‐specific reproductive and somatic allocation may more generally underscore plasticity within this trade‐off since other environmental stressors result in similar patterns of allocation (Auld & Houser, 2015; Bascunan‐Garcia, Lara, & Cordoba‐Aguilar, 2010; Kaitala, 1991; Tanaka & Suzuki, 1998; Visanuvimol & Bertram, 2010). For example, similar findings have been observed when insect species, including *A. domesticus,* are faced with immune challenges (Adamo, 1999; Bascunan‐Garcia et al., 2010; Sadd et al., 2006). Though, not all organisms respond this way to aging (Cotter et al., 2010; Nussey et al., 2008), it would appear that house crickets switch toward reproducing at a high rate when they begin producing offspring at an advanced age or are presented with challenges that indicate limited future reproduction, which adversely affects their subsequent lifespans (e.g., immune challenge).

Studies have shown that female house crickets prefer large males (Gray, 1997; Ryder & Siva‐Jothy, 2001; Stoffer & Walker, 2012 but see: Mautz & Sakaluk, 2008) and male size can be considered an indicator of male quality (Gray, 1997; Ryder & Siva‐Jothy, 2001; Stoffer & Walker, 2012). Here, we find that in addition to being important for mate choice, male size also has a role in sexual conflict. Sexual conflict is an important factor in shaping life history trade‐offs (Brooks & Garratt, 2017; Chapman, 2006; Chapman, Arnqvist, Bangham, & Rowe, 2003; Johnstone & Keller, 2000), and here, we found that male size had a significant impact on female reproductive and somatic allocation patterns. Females mating with smaller, presumably nonpreferred, males had decreased postmating and total lifespans and increased egg‐laying rates irrespective of female age at first mating. These patterns suggest male manipulation and/or harassment of females by nonpreferred males, which is consistent with sexual conflict theory. Indeed, similar trends of sexual conflict have been previously described in crickets as well as other insects and involve manipulating females through ejaculate contents (Garcia‐Gonzalez & Simmons, 2010; Green & Tregenza, 2009; Pitnick & García‐González, 2002; South & Lewis, 2011) indicating male house crickets could be capable of this strategy (Fleischman & Sakaluk, 2004). Wagner and Harper (2003) observed a similar pattern in the variable field cricket in relation to preferred male calling characteristics, female reproduction, and lifespan and suggest that female preferences may select males with beneficial ejaculates. Since female house crickets prefer large males and mating with these males resulted in less manipulation (e.g., longer postmating lifespans) a similar correlation would be expected here.

Studies in crickets have not identified patterns of phenotype‐correlated male manipulation through male seminal fluids. However, these correlations have been found with sperm number (Klaus, Fitzsimmons, Pitcher, & Bertram, 2011; Wagner & Harper, 2003) and males can differentially allocate sperm based on environmental cues (Bailey, Gray, & Zuk, 2010) including the characteristics of his mate (Thomas & Simmons, 2007). Specifically, in house crickets, a negative relationship between male house cricket mass and the number of sperm transferred in spermatophores has been observed (Klaus et al., 2011), although the effect of differential sperm transfer on female laying rate remains to be studied. Thus, it is likely that male effects on female life history traits resulted from some degree of female manipulation via spermatophore contents or behavioral interactions during the time they were paired. Increased egg‐laying rate, along with reduced lifespans, observed for females exposed to small males may be associated with higher levels of manipulation through spermatophore contents. While the relationship between male size and seminal fluid content has not been studied in house crickets, male house crickets can manipulate females through the transfer of prostaglandins during mating, which increases female egg‐laying rates (Larson et al., 2012; Murtaugh & Delinger, 1987; Worthington et al., 2015) presumably at the cost of her longevity (South & Lewis, 2011; Zajitschek, Lailvaux, Dessmann, & Brooks, 2012). Given their presence in seminal fluids across taxa (Kennedy, Korn, & Thurston, 2003; Templeton, Cooper, & Kelly, 1978; Worthington et al., 2015), prostaglandins may play a role in sexual conflict across taxa (Sirot, Wong, Chapman, & Wolfner, 2015). The role of prostaglandins in cricket reproduction is complicated since they are depleted after mating. Worthington et al. (2015) hypothesize female *G. texensis* who re‐mate have higher fecundity, in part, due to increased prostaglandin levels which stimulate egg laying. Thus, in crickets, it is unclear if prostaglandins/accessory substances manipulate females or play a key role as a gift that stimulates egg laying (Green & Tregenza, 2009; Worthington et al., 2015).

Interestingly, larger or more dominant male insects tend to cause more harm to females (Bretman et al., 2006; Iglesias‐Carrasco et al., 2018; Pitnick & García‐González, 2002) while results here suggest that smaller house crickets may cause more harm through female manipulation, which aligns with the idea that preferred males may transfer more beneficial (or less costly) ejaculates (Wagner & Harper, 2003). If it is costly for males to alter female investment patterns, then a trade‐off between male body size and mate manipulation may exist. Since small males are not preferred by females, these males are likely to obtain fewer matings and would benefit from tactics that help them obtain more offspring from each mating despite the costs associated with these tactics. In addition to indicating better immune response (Ryder & Siva‐Jothy, 2001), larger body size may also indicate to a potential mate that he will inflict less harm. Studies aimed at isolating direct and indirect benefits/costs associated with male quality will allow us to better understand the role of sexual conflict in female preference and male trade‐offs.

While females may postpone mating if a high‐quality mate is not available (Gray, 1999; Moore & Moore, 2001; Wilgers & Hebets, 2012), this strategy could be detrimental given that females postponing reproduction produced fewer eggs regardless of the size of their mate. There are potentially strong indirect and possibly direct benefits from mating with a high‐quality male when other fitness components are considered (Garcia‐Gonzalez & Simmons, 2010; Head, Hunt, Jennions, & Brooks, 2005). Thus, it is possible these benefits could offset the reduced fecundity observed for intermediate and old females exposed to large males across multiple generations and contribute to delayed mating as a viable reproductive strategy.

## CONFLICT OF INTEREST

None declared.

## AUTHOR CONTRIBUTIONS

Both authors conceived and designed the experiment and wrote the manuscript. KMW performed the experiment and SEW analyzed the data.

## Data Availability

Data for this study have been archived at figshare: http://dx.doi.org/10.6084/m9.figshare.1209125
